# Identifying the barriers and facilitators to fruit and vegetable consumption in rural Australian adults: a mixed methods analysis

**DOI:** 10.1186/s12937-024-00972-y

**Published:** 2024-06-28

**Authors:** Brooke T. Carroll, Sarah A. McNaughton, Kate E. Parker, Laura E. Marchese, Katherine M. Livingstone

**Affiliations:** 1 Institute for Physical Activity and Nutrition, School of Exercise and Nutrition Sciences, Deakin University, Geelong, VIC, 3220 Australia; 2 Health and Well-Being Centre for Research Innovation, School of Human Movement and Nutrition Sciences, University of Queensland, St Lucia, QLD 4067 Australia; 3 City of Greater Bendigo, Active and Healthy Communities, Bendigo, VIC 3552 Australia; 4 Institute for Physical Activity and Nutrition, School of Exercise and Nutrition Sciences, Deakin University, Melbourne Burwood Campus, 221 Burwood Highway, Melbourne, VIC 3220 Australia

## Abstract

**Background:**

Low fruit and vegetable consumption is a leading contributor to non-communicable disease risk. However, understanding of barriers and facilitators to fruit and vegetable intake in rural settings is limited. This study used a mixed methods approach to determine the barriers and facilitators to increasing fruit and vegetable intake in rural Australian adults and to identify if these varied by gender.

**Methods:**

Quantitative and qualitative data were used from the 2019 Active Living Census, completed by adults living in north-west Victoria, Australia. Data were collected on fruit and vegetable intakes and barriers and facilitators to meeting fruit and vegetable recommendations. Multivariate logistic regression analyses were used to investigate the association between facilitators, classified using the socio-ecological framework, and meeting recommendations. Machine learning was used to automate content analysis of open ended information on barriers.

**Results:**

A total of 13,464 adults were included in the quantitative analysis (51% female; mean age 48 [SE 0.17] years) with 48% and 19% of participants consuming the recommended two serves of fruit and five serves of vegetables daily, respectively. Strongest facilitators to fruit consumption were at the individual level: never smoked (OR: 2.12 95% CI: 1.83–2.45) and not drinking alcohol (OR: 1.47 95% CI: 1.31–1.64). Strongest facilitators for vegetable consumption were found at all levels; i.e., individual level: used to smoke (OR: 1.48 95% CI: 1.21–1.80), social-environmental level: living with three or more people (OR: 1.41 95% CI: 1.22–1.63), and physical-environmental level: use community gardens (OR: 1.20 95% CI: 1.07–1.34). Qualitative analyses (fruit *n* = 5,919; vegetable *n* = 9,601) showed that barriers to fruit consumption included a preference for other snacks and desire to limit sugar content, whilst lack of time and unachievable guidelines were barriers for vegetables. Barriers and facilitators differed by gender; females experienced barriers due to having a more varied diet while males reported a dislike of the taste.

**Conclusions:**

Barriers and facilitators to fruit and vegetable consumption among rural Australian adults were identified across all levels of the socio-ecological framework and varied between fruit and vegetables and by gender. Strategies that address individual, social, and physical-level barriers are required to improve consumption.

**Supplementary Information:**

The online version contains supplementary material available at 10.1186/s12937-024-00972-y.

## Background

Non-communicable diseases (NCDs), including cardiovascular disease, cancer, and diabetes, are a leading cause of early death globally [1], and contribute to 89% of early deaths in Australia [1]. However, the burden of disease is disproportionately distributed across major cities and rural areas [2], with 53% of rural Australians living with a NCD compared with 46% in major cities [3]. Dietary risk factors, including low fruit and vegetable consumption, are a significant concern and contribute to 9.9% of deaths in Australia [4–7]. Within rural areas, 58% of Australians are not meeting the recommended two serves of fruit per day and 96% are not meeting the recommended five serves of vegetables per day [2]. Increasing fruit and vegetable intake within rural Australia may help reduce the disproportionate health burden experienced by these communities [6, 7].

The barriers and facilitators to fruit and vegetable consumption include a wide range of determinants [8]. The socio-ecological framework is a useful tool for understanding such determinants, as it constitutes three broad levels: individual (a person’s cognitive, biological and demographic characteristics), social-environmental (how a person interacts with their social environment) and physical-environmental (how a person interacts with their natural and built environment) [9]. At the individual level, previous research suggests that being female, older in age, at a higher socio-economic status and greater food security are facilitators to fruit and vegetable consumption [10–18]. Similarly, not living alone (social-environmental) [19–22] and using community gardens (physical-environmental) [23–25] have also been identified as facilitators. Moreover, commonly barriers to meeting fruit and vegetable recommendations include a lack of time, high cost and limited access to fresh produce [12, 26]. However, literature is limited by a small number of studies completed in rural settings [27] and available studies having a small sample size [20, 28]. Furthermore, investigation of barriers to fruit and vegetables separately will inform the design of targeted strategies that may lead to more effective interventions. Yet few studies have done so [29]. Thus, there is a need to investigate in a larger sample size, from rural Australia with stratification by the two food groups. Additionally, research suggests that determinants of fruit and vegetable intake differ by gender, [12] but studies rarely stratify determinants by this [13, 30]. Therefore, the aim of this study was to identify the barriers and facilitators to increasing fruit and vegetable consumption in rural Australian adults and whether these varied by gender.

## Methods

### Study design and participants

This study involved secondary data analysis of the 2019 Active Living Census, which was conducted in the Loddon Campaspe region of north-west Victoria, Australia. All households in the Loddon Campaspe region (*n* = 224,947) were invited to participate between 20 May 2019 and 16 June 2019 [31]. Households were mailed a census booklet that contained a hard copy questionnaire and an invitation to complete an online version. Incentives for census completion were offered and included vouchers for the supermarket and local bike and sport stores. Completion of the census was also promoted using an integrated advertising campaign on local radio, television, print and social media. The census included questions on socio-demographic characteristics, health and well-being, financial situation, physical activity and the use of public and open spaces/facilities (Additional File S3) [31].

A mixed methods approach was used to accommodate closed and open-ended questions. The STROBE-nut and COREQ checklists were used for reporting quantitative and qualitative data, respectively (Additional File S1 and S2) [32, 33]. Participants were excluded from the quantitative analysis if (i) they were < 18 years of age, (ii) they had missing data for exposure or outcome variables, (iii) fruit or vegetable intake was considered implausible (more than three standard deviations above or below the mean), [34, 35], or (iv) reported height and/or weight were considered implausible (outside of ranges detailed below), [36] or (v) the recorded postcode fell outside the Loddon Campaspe Region [37]. For the qualitative analysis participants were excluded if (i) they were < 18 years of age, (ii) they had missing data for the relevant outcome variables, or (iii) a response was not relevant for analysis (e.g., “don’t know”, “unsure”, “no reason”). An ethics exemption for analysis of existing data was granted by the Deakin University Human Research Ethics Committee (Reference number 2023-095).

### Study measures

#### Fruit and vegetable intake

Participants were asked to report the number of servings of vegetables (inclusive of legumes and beans) and fruit they consumed each day. These questionnaire items were adapted from the 2015 VicHealth Indicators Survey and were originally designed by the Australian Bureau of Statistics [2]. To inform participants of what constitutes a serve of fruit and vegetables, information from the Eat for Health Guidelines were included in the questionnaire (i.e. one serve of vegetables equates to half cup of cooked vegetables or one cup of salad, while one serve of fruit equates to one medium or two small pieces of fruit or one cup of diced fruit) [5].

#### Facilitators

Information from quantitative data were used to identify facilitators to fruit and vegetable intake, which were classified according to the socio-ecological framework: individual, social-environmental and physical-environmental determinants [9, 38]. A list of questionnaire items and response options that were used in this analysis are provided in Additional File S3.

At the individual level, information was collected on age, gender identity, household financial stability and highest level of education. Area level disadvantage was determined by matching postcodes to the Australian Bureau of Statistics 2016 Socio-Economic Indexes for Areas (SEIFA) using the Index of Relative Socio-Economic Disadvantage and was categorised into quartiles ranging from the most disadvantaged (quartile 1) to the least disadvantaged (quartile 4) [39]. Health behaviours were assessed using questionnaire items on smoking status, alcoholic beverage intake and sugar sweetened drink consumption. The questionnaire also included items on water consumption, physical activity, height, and weight. Finally, information on household size and household food insecurity (social-environmental) as well as use of community gardens (physical-environmental) was collected.

#### Barriers

Information on barriers to fruit and vegetable intake was collected by asking participants to state the main reason why they did not meet the recommended intakes of both fruit and vegetables [5]. These two questions were preceded by a short statement explaining the recommended intake; “Health experts say that you should eat at least 5 serves of vegetables a day” and “Health experts say that you should eat at least 2 serves of fruit a day” [31].

### Quantitative analysis

Quantitative analysis of fruit and vegetable intake and facilitators was undertaken using Stata software (version 18; StataCorp., College Station, TX, USA). A complete case analysis was used. A comparison of participants characteristics in the excluded and analytic sample is presented in Additional File 4. No responses were three standard deviations above or below the mean for both fruit and vegetable intake. Descriptive statistics for continuous variables (mean and standard error [SE]) and categorical variables (frequencies [%]) were used.

Univariate and multivariate logistic regression analyses were utilised to determine odds ratios (OR) and 95% confidence intervals (CI) of participants meeting fruit and vegetable recommendations (dependent variables) according to the individual, social-environmental and physical-environmental determinants (independent variables). Sampling error was partially controlled for by using a weighting variable, which weighted data to Australian Bureau of Statistics benchmarks by age, education, gender, and country of birth [31]. Analyses were stratified by gender (female and male). Due to a small sample, gender diverse data were used for descriptive purposes only. Independent variables were included in the multivariate logistic regression model if the likelihood-ratio test from the univariate model was *p* < 0.05 [40]. In the multivariate models, independent variables were considered to statistically influence odds of meeting fruit and vegetable recommendations if *p* < 0.05 [40, 41]. Multicollinearity was assessed using the variance inflation factor to ensure independent variables were not highly correlated. No evidence of multicollinearity was observed (variance inflation factor < 5).

### Quantitative analysis

Analysis of open-ended responses to barriers were undertaken using Leximancer software version 5 (Leximancer Pty Ltd). This software uses machine learning to automate content analysis and creates a visual representation of concepts. Leximancer shows face validity, stability and reproducibility, therefore, is regarded as a valid and reproducible method of qualitative analysis [12, 42].

Spelling errors were corrected by the main author (BTC), and responses deemed not relevant for analysis (e.g., ‘don’t know’, ‘no reason’, ‘?’) were removed prior to the responses being imported into Leximancer. Leximancer used a semantic extraction phase to define concepts (groups of words that appear together within the text), and a thesaurus was generated for each concept. These concepts were displayed as dots on a concept map, where the closer proximity of concepts on the map infers that they were often mentioned together in the text. Concepts were further grouped by the main author (BTC) into themes if they were highly connected and were displayed as coloured circles on the concept map. The colours represented relevance of themes; warmer colours (red, yellow) were more relevant (more frequent within the text) than cooler colours (green, blue, purple). A smaller theme circle size included a smaller number of concepts, and a larger theme circle size included a larger number of concepts. The themes and theme sizes were examined for interpretability by two additional researchers (KML and SAM). Quotes were extracted from Leximancer by main author (BTC) to best represent each theme, they were verified by an additional researcher (KML).

The majority of Leximancer’s default settings were utilised. Sentences per block was changed to one as most survey responses were one sentence long. Identifying name-like concepts (the identification of concepts through upper case words) were switched off as proper nouns were not relevant for this analysis [43]. The stop words (words not available for analysis) ‘eat’ and ‘like’ were removed to ensure they could be available for analysis; [43] the stop word ‘don’t’ was added. Similar concepts that appeared in close proximity on the concept map were merged (e.g., ‘snack’ and ‘snacks’; ‘eat’ and ‘eating’). Words ‘fruit’ and ‘vegetables’ were omitted from their respective concept maps due to their frequent use and to ensure they did not feature as concepts. Themes were renamed across all concept maps to provide a better description of the concepts within each theme. The top three themes from each concept map were analysed due to minimal relevance of the less prominent themes.

## Results

### Participant characteristics

**Fig. 1:**
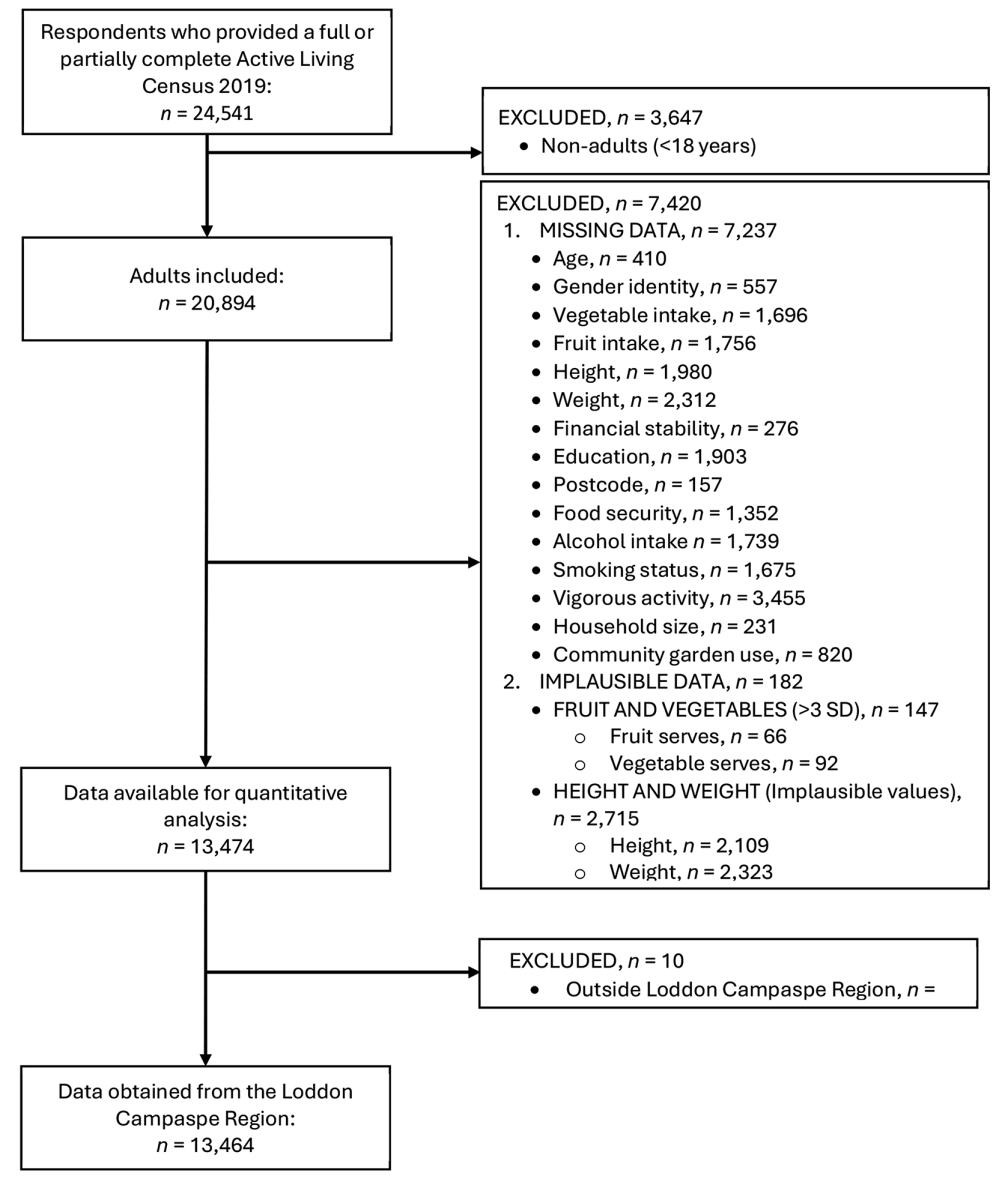
Flow diagram of exclusion criteria for Active Living Census 2019 participants for quantitative analysis

**Fig. 2:**
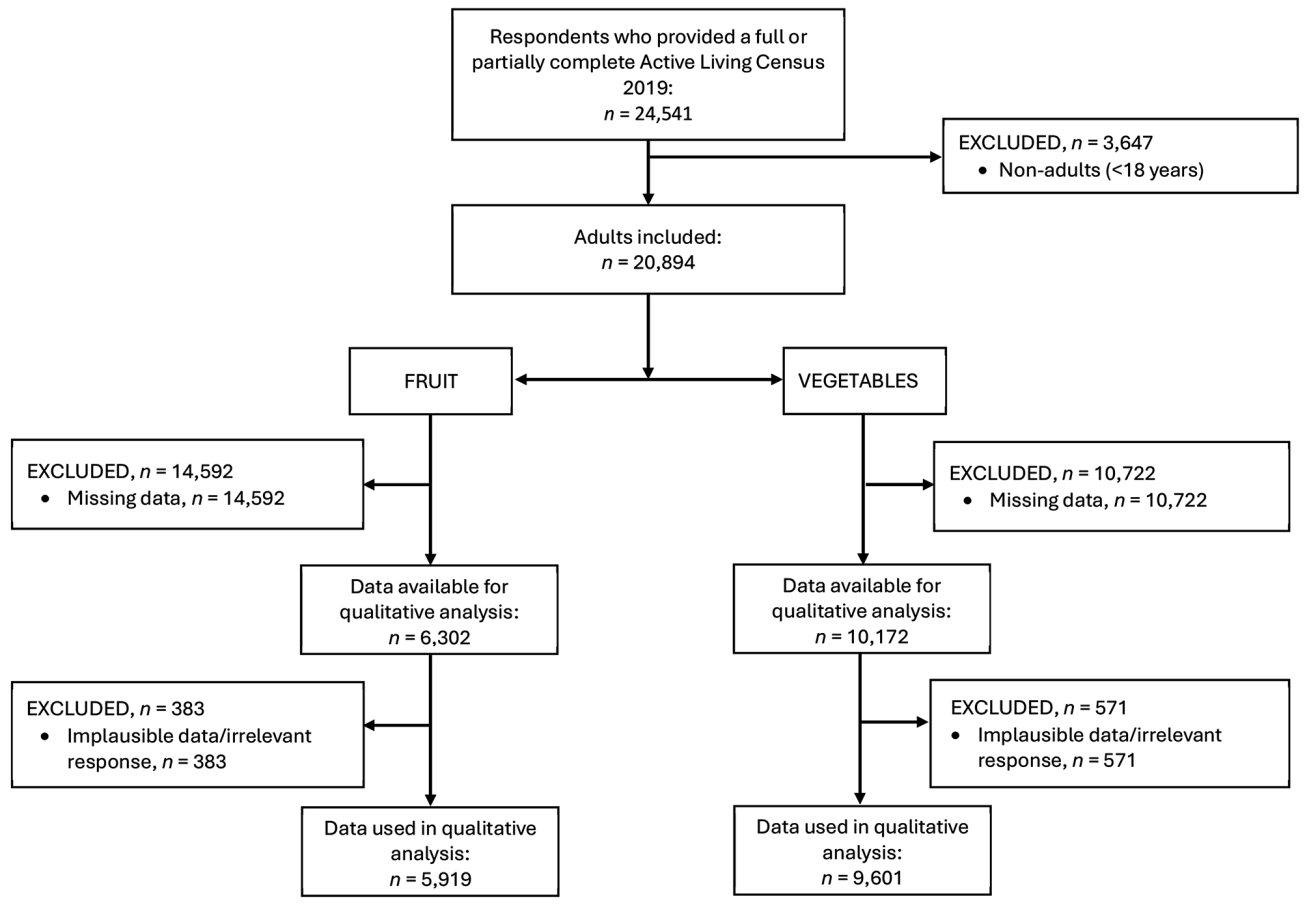
Flow diagram of exclusion criteria for Active Living Census 2019 participants for qualitative analysis

Of the 224,947 adults and children within the Loddon Campaspe region, a total of 24,541 responses (14,473 hard copy and 10,068 soft copy) were returned from 13,524 households. Therefore, the response rate (i.e., number of forms received as a proportion of forms mailed) was 10.9%. Figures 1 and 2 show the excluded data for the quantitative and qualitative analysis respectively.

The socio-demographic characteristics of the participants are shown in Table 1. The mean age was 48.0 (SE 0.17) years overall, and 51% were female. The mean body mass index (BMI) was 27.4 (SE 0.06) kg/m^2^, with 35.5% classified as overweight and 26.3% as having obesity. Compared with females, males were on average older (male: mean 48.8, SE 0.27 years; female: mean 47.3, SE 0.22 years), with the majority aged 51–70 years (34.7%) (females were mostly aged between 31 and 50 years; 35.9%). Most males were overweight (42.4%) with a mean BMI of 27.5 (SE 0.08) kg/m^2^. In contrast, females were mostly underweight/normal weight (43.0%), however average BMI (mean: 27.4, SE 0.01 kg/m^2^) was comparable between genders. Comparted to the analytic sample, the excluded sample had higher prevalence of obesity and were older (Additional File S4).

**Table 1:** Fruit and vegetable intake and socio-demographic and anthropometric characteristics overall and by gender from the active living Census 2019 (*n* = 13,464)

Characteristic	Overall^1^ (*n* = 13,464)	Male^1^ (*n* = 5771)	Female^1^ (*n* = 7662)
Meet fruit recommendations, n (%)^2^	7042 (52.3)	2695 (46.7)	3724 (48.6)
Meet vegetable recommendations, n (%)^2^	6422 (18.5)	1050 (18.2)	1448 (18.9)
Age (years), mean (SE)	48.0 (0.17)	48.8 (0.27)	47.3 (0.22)
Age groups, %			
18–30 years	2599 (19.3)	1068 (18.5)	1154 (20.0)
31–50 years	4739 (35.2)	1985 (34.4)	2072 (35.9)
51–70 years	4685 (34.8)	2003 (34.7)	2014 (34.9)
> 70 years	1441 (10.7)	716 (12.4)	529 (9.17)
Gender, n (%)			
Male	6557 (48.7)	-	-
Female	6867 (51.0)	-	-
Gender diverse	31 (0.23)	-	-
Height (cm), mean (SE)	171.3 (0.11)	178.2 (0.13)	164.6 (0.10)
Weight (kg), mean (SE)	80.6 (0.19)	87.3 (0.26)	74.1 (0.24)
BMI (kg/m^2^), mean (SE)	27.4 (0.06)	27.5 (0.08)	27.4 (0.09)
Weight status, n (%)^3^			
Underweight/Normal weight (< 25 kg/m^2^)	5157 (38.3)	1922 (33.3)	2482 (43.0)
Overweight (25–30 kg/m^2^)	4780 (35.5)	2447 (42.4)	1668 (28.9)
Obesity (> 30 kg/m^2^)	3528 (26.3)	1402 (24.3)	1627 (28.2)

1, Values represented weighted mean and standard errors (SE) for continuous variables and weighted frequencies for categorical variables. 2, Recommended intake of fruit and vegetables was 2 and 5 serves/day, respectively. 3, Weight status categories were determined based on BMI cut offs from the World Health Organisation [44]

#### Fruit and vegetable intake and determinants

The mean consumption of fruit was 1.56 (SE 0.01) serves per day with males consuming 1.54 (SE 0.02) and females consuming 1.58 (SE 0.01) serves per day. The mean vegetable intake was 2.85 (SE 0.02) serves per day with males consuming 2.76 (SE 0.03) and females consuming 2.94 (SE 0.02) serves per day. The proportion of participants who met recommended intakes of fruit and vegetables was higher in females than males (Table 1).

#### Facilitators to fruit and vegetable consumption

The odds of meeting recommendations for fruit intake and for vegetable intake according to each determinant are presented in Table 2. Results from the multivariate models suggest that facilitators of meeting fruit recommendations (Table 2) for all genders at the individual level were being older in age, more financially stable, obtaining a tertiary degree, being underweight/normal weight, drinking alcohol less frequently, being a non-smoker, completing more vigorous physical activity. Being more food stable was identified as a facilitator of meeting fruit recommendations at the social-environmental level, and use of community gardens was identified as a facilitator at the physical-environment level. When stratified by gender, food stability was not significantly associated with odds of meeting fruit recommendations. Financial stability was not significantly associated with odds of meeting fruit recommendations in males or females.

**Table 2:** Odds of meeting recommended fruit and vegetable intake in adults from the active living Census 2019 (*n* = 13,464)

Characteristic	Fruit^1^			Vegetable^1^		
	Overall	Male	Female	Overall	Male	Female
	OR (95% CI)	OR (95% CI)	OR (95% CI)	OR (95% CI)	OR (95% CI)	OR (95% CI)
**Individual determinants**						
Gender						
Male	1.0			1.0	-	-
Female	1.07 (0.99–1.16)			1.16 (1.06–1.27)	-	-
Gender Diverse	0.95 (0.46–1.96)			1.53 (0.68–3.47)	-	-
Age	1.02 (1.01–1.02)	1.01 (1.01–1.02)	1.02 (1.01–1.02)	1.01 (1.01–1.02)	1.01 (1.00-1.01)	1.02 (1.01–1.02)
Financial stability						
Prosperous/Very comfortable	1.0	1.0	1.0	1.0	1.0	1.0
Reasonably comfortable	0.91 (0.83–0.99)	0.91 (0.79–1.05)	0.90 (0.80–1.03)	0.86 (0.78–0.97)	0.84 (0.71–0.99)	0.88 (0.76–1.02)
Just getting along/Poor/Very poor	0.77 (0.69–0.87)	0.74 (0.62–0.88)	0.79 (0.68–0.93)	0.81 (0.71–0.93)	0.82 (0.67–1.02)	0.80 (0.66–0.96)
Education level						
Tertiary degree or higher	1.0	1.0	1.0	1.0	1.0	1.0
Completed year 12	0.82 (0.75–0.89)	0.79 (0.69–0.89)	0.83 (0.75–0.92)	0.77 (0.70–0.86)	0.85 (0.73–0.99)	0.71 (0.63–0.82)
Not completed year 12	0.83 (0.75–0.92)	0.85 (0.74–0.98)	0.78 (0.68–0.90)	0.84 (0.74–0.95)	0.98 (0.82–1.17)	0.71 (0.60–0.84)
Area level disadvantage						
1 – most disadvantaged	1.0	1.0	1.0	1.0	-	1.0
2	1.07 (0.95–1.19)	1.05 (0.89–1.26)	1.07 (0.93–1.24)	1.10 (0.95–1.27)	-	1.08 (0.90–1.31)
3	1.25 (1.07–1.47)	1.36 (1.06–1.74)	1.17 (0.95–1.45)	1.30 (1.07–1.58)	-	1.28 (0.99–1.64)
4	1.09 (0.96–1.23)	1.12 (0.93–1.35)	1.05 (0.90–1.24)	1.10 (0.94–1.29)	-	1.11 (0.90–1.36)
5 – least disadvantaged	1.12 (0.97–1.30)	1.28 (1.02–1.60)	0.99 (0.81–1.20)	1.20 (1.00-1.43)	-	1.20 (0.95–1.52)
Weight status^2^						
Underweight/Normal weight	1.0	1.0	1.0	1.0	1.0	1.0
Overweight	0.83 (0.76–0.90)	0.83 (0.73–0.94)	0.83 (0.74–0.92)	0.85 (0.77–0.94)	0.82 (0.71–0.96)	0.87 (0.76–0.99)
Obesity	0.70 (0.64–0.77)	0.76 (0.65–0.88)	0.67 (0.59–0.75)	0.73 (0.65–0.82)	0.73 (0.61–0.88)	0.71 (0.62–0.83)
Smoking status						
Current smoker	1.0	1.0	1.0	1.0	1.0	1.0
Used to smoke	1.98 (1.71–2.29)	1.97 (1.59–2.44)	2.04 (1.66–2.51)	1.48 (1.21–1.80)	1.43 (1.07–1.89)	1.58 (1.20–2.08)
Never smoked	2.12 (1.83–2.45)	2.17 (1.76–2.68)	2.10 (1.72–2.57)	1.43 (1.17–1.73)	1.39 (1.05–1.83)	1.51 (1.15–1.98)
Alcoholic beverage intake						
More than 3 days per week	1.0	1.0	1.0	1.0	-	1.0
1–2 days per week	1.35 (1.22–1.50)	1.33 (1.15–1.53)	1.37 (1.28–1.58)	1.08 (0.96–1.22)	-	1.05 (0.88–1.24)
3 or less days per month	1.35 (1.22–1.48)	1.26 (1.09–1.45)	1.45 (1.27–1.66)	1.01 (0.90–1.14)	-	1.01 (0.87–1.19)
No longer drink/do not drink	1.47 (1.31–1.64)	1.36 (1.15–1.63)	1.57 (1.35–1.83)	1.15 (1.00-1.32)	-	1.15 (0.96–1.37)
Vigorous physical activity (hours per week)	1.05 (1.04–1.06)	1.04 (1.03–1.06)	1.06 (1.05–1.08)	1.04 (1.03–1.05)	1.03 (1.02–1.05)	1.05 (1.03–1.06)
**Social-environmental determinants**						
Household size						
1 person	1.0	1.0	1.0	1.0	1.0	1.0
2 people	1.14 (1.03–1.26)	1.26 (1.06–1.49)	1.12 (0.98–1.27)	1.40 (1.23–1.60)	1.86 (1.46–2.38)	1.27 (1.09–1.49)
3 + people	1.07 (0.96–1.20)	1.19 (0.98–1.43)	1.04 (0.90–1.20)	1.41 (1.22–1.63)	1.83 (1.40–2.39)	1.28 (1.07–1.54)
Food stability						
Food unstable	1.0	1.0	1.0	1.0	1.0	1.0
Food stable	1.19 (1.02–1.40)	1.09 (0.84–1.42)	1.25 (1.03–1.54)	1.29 (1.04–1.61)	1.65 (1.12–2.44)	1.14 (0.87–1.50)
**Physical-environmental determinants**						
Use of Community Gardens						
Haven’t used facility	1.0	1.0	1.0	1.0	-	1.0
Have used facility	1.18 (1.10–1.27)	1.18 (1.05–1.32)	1.19 (1.08–1.31)	1.15 (1.06–1.26)	-	1.20 (1.07–1.34)

1, Values represented weighted odds ratios (OR) and 95% confidence intervals (CI) for meeting vegetable recommendations. The recommended vegetable intake was considered more than five serves per day. The recommended fruit intake was considered more than two serves per day [5]. Multivariate models include all facilitators that were determined to be statistically significant (*p* < 0.05) in the univariate models. 2, Weight status categories were determined based on BMI cut offs from the World Health Organisation [44]

In the multivariate model, facilitators of meeting vegetable recommendations (Table 2) were found across all levels of the socio-ecological framework, i.e., being female, older, financially prosperous, completing a tertiary degree or higher, underweight/normal weight, a non-smoker, completing more vigorous physical activity (individual level), living with others, being food stable (social-environmental level) and using community gardens (physical-environmental level). In men, being prosperous/very comfortable was a facilitator of meeting vegetable intake recommendations compared to those who are reasonably comfortable, but not compared to those who are just getting along/poor/very poor. While for women, being prosperous/very comfortable was a facilitator of meeting vegetable intake recommendations compared to those who were just getting along/poor/very poor, but not compared to those who were reasonably comfortable. For education, in men, having a tertiary degree was a facilitator of meeting vegetable intake recommendations compared to those who completed year 12, but not compared to those who did not complete year 12. In women, higher education was a facilitator of vegetable consumption, regardless of the level.

#### Barriers to fruit and vegetable consumption

The themes and related concepts for the barriers to meeting the daily fruit and vegetable intake recommendations in the overall sample are reported in Additional File 5. Four themes were identified for fruit, these were, in decreasing importance: Eat, Time, Sugar and Seasonal. To aid interpretation, these barriers for fruit were renamed to (1) Preference and appetite; (2) Cost, quality, and time constraints; (3) Sweetness and diet; (4) Seasonality. Six themes were identified for vegetables: Time, Eat, Diet, Meat, Cost and Appetite; stated from most to least important. These barriers for vegetables were renamed to (1) Cost, quality, and time constraints (merged with theme 5, cost); (2) Preference and appetite (merged with theme 6, appetite); (3) Satisfied with current diet; (4) Preference for meat. For simplicity, the renamed themes will be used from here onwards to discuss the barriers.

#### Barriers to fruit consumption by gender

**Fig. 3:**
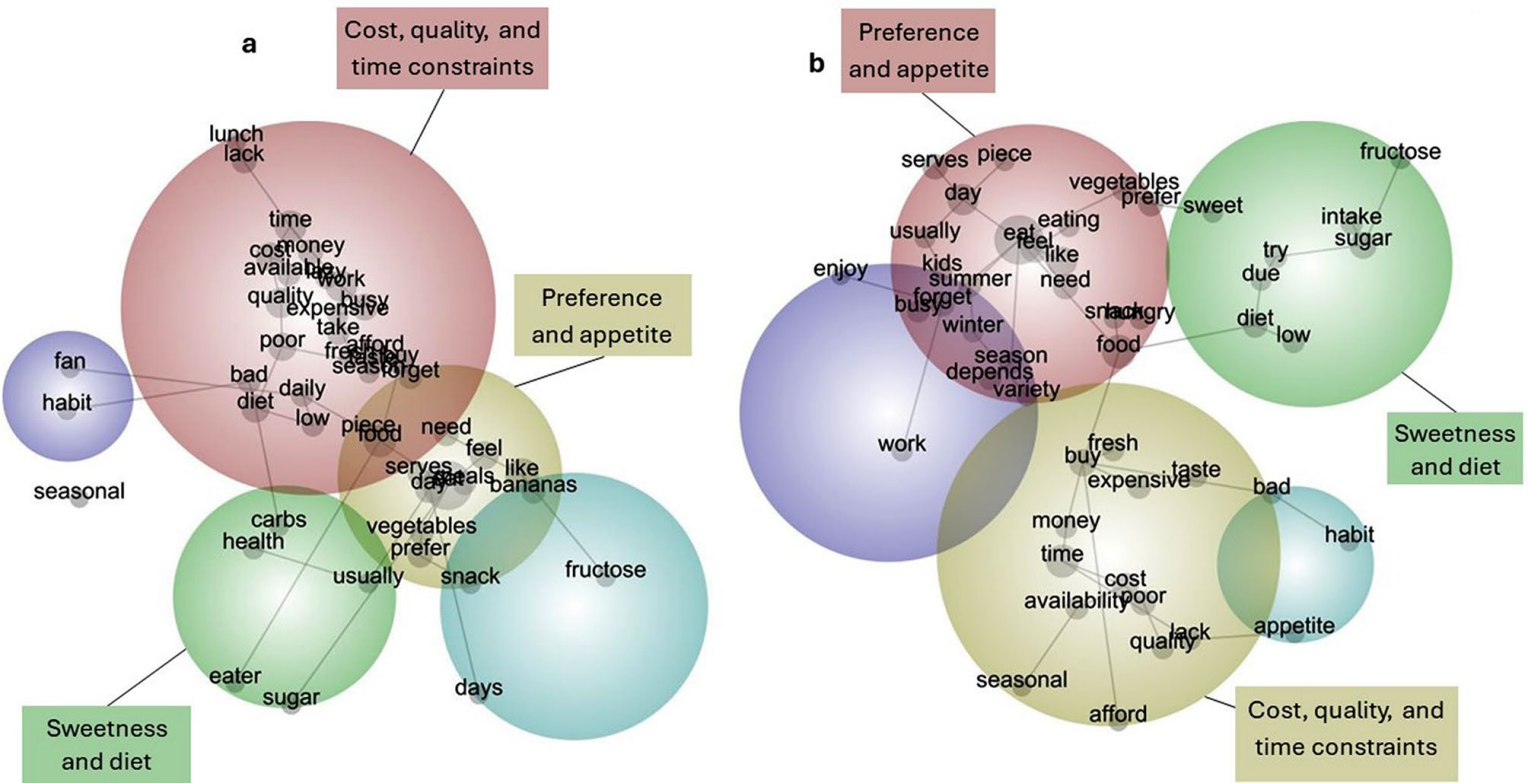
Barriers to fruit consumption for the Active Living Census 2019 in (**a**) males; (**b**) females

In males, barriers to fruit consumption were (1) cost, quality, and time constraints and (2) preference and appetite. For females, the most important barriers were (1) preference and appetite, (2) cost, quality, and time constraints and (3) sweetness and diet. (Fig. 3; Table 3).

**Table 3:** Themes and concepts for barriers to fruit consumption by gender from the Active Living Census 2019 (*n* = 5,919)

Theme	Male & Female		Male		Female	
	Concept	Quote	Concept	Quote	Concept	Quote
**Preference and appetite**	Like	“Don’t feel like it.” (Participant 8396, 48 years)	Prefer	“Prefer other processed foods to snack on.” (Participant 2292, 47 years)	Forget	“Most days I would have 2 serves, but on the days I have 1 serve it’s because I forget.” (Participant 2164, 58 years)
	Day	“I like one piece a day.” (Participant 6688, 56 years)			Feel	“Don’t always feel like it.” (Participant 10,182, 72 years)
					Serves	“It varies. Sometimes I can eat over 2 serves and other days none at all. Just depends what I have as a snack.” (Participant 6503, 23 years)
**Cost, quality, and time constraints**	Cost	“Cost from local supermarket is excessive & quality is bad.” (Participant 7361, 42 years)	Work	“Not enough time at work.” (Participant 2488, 24 years)	Buy	“Expensive also they go off when I don’t feel like them so I don’t buy them often.” (Participant 6006, 21 years)
			Lack	“Lack of time to prepare lunch in the morning.” (Participant 9955, 28 years) “Lack of money.” (Participant 10,269, 46 years)		
			Busy	“Often feel too busy to properly prepare meals and snacks for the week.” (Participant 192, 29 years)		
**Sweetness and diet**	Sugar	“Trying to limit sugar intake.” (Participant 1736, 42 years)	Diet	“I feel my diet is adequate.” (Participant 4019, 77 years) “Following low carb diet.” (Participant 14,203, 65 years)	Diet	“I’m on a keto diet - cannot consume carbohydrates” (Participant 5442, 20 years)

Consistent and divergent barriers and the corresponding concepts across genders are summarised in Table 3. For the barrier “preference and appetite”, both males and females reported the belief that one piece of fruit was enough, males stated they preferred other snacks and females felt that their serving consumption varied per day. Both genders perceived that the cost of fruit was too high, reflected in the “cost, quality, and time constraints” theme. Additionally, males reported they were too busy (often due to work), and females stated that fresh produce went off too quickly. Finally, in the “sweetness and diet” theme the overall sample expressed a preference to limit their sugars intake, with some females indicating they were following a specific diet that deterred them from eating fruit.

#### Barriers to vegetable consumption by gender

**Fig. 4:**
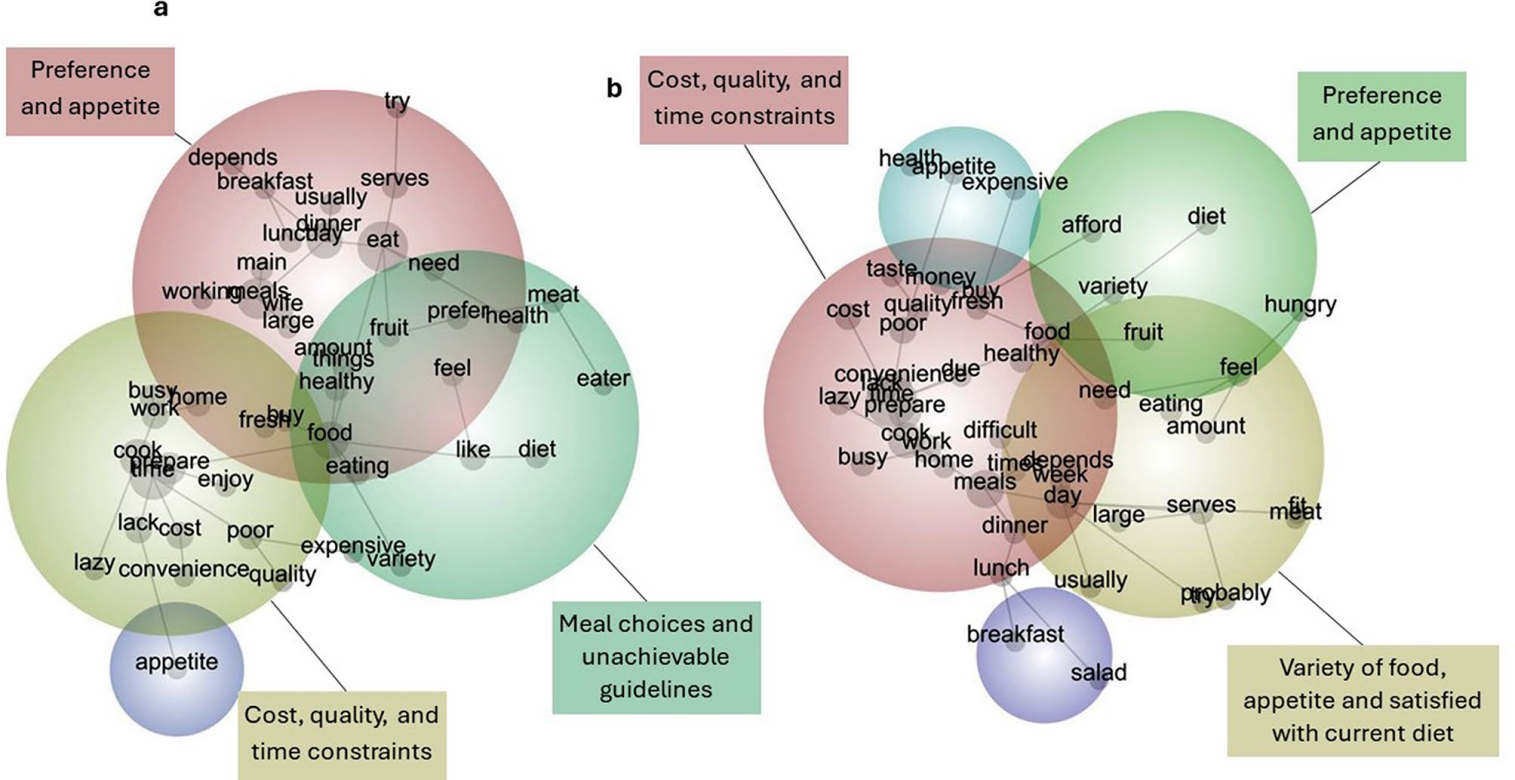
Barriers to vegetable consumption for the Active Living Census 2019 in (**a**) males; (**b**) females

The three most important barriers to meeting vegetable recommendations in males were (1) preference and appetite, (2) cost, quality, and time constraints and (3) meal choices and unachievable guidelines (Fig. 4; Table 4). Barriers in females were (1) cost, quality, and time constraints, (2) preference and appetite and 3) variety of food, appetite and satisfied with current diet (Fig. 4; Table 4).

**Table 4:** Themes and concepts of barriers to vegetable consumption from the Active Living Census 2019 (*n* = 9,601)

Theme	Male & Female		Male		Female	
	Concept	Quote	Concept	Quote	Concept	Quote
**Cost, quality, and time constraints**	Cook	“Cost and time and energy to cook and plan meals.” (Participant 5588, 36 years)			Lack	“Lack of time at work to eat sufficiently.” (Participant 5970, 26 years) “Lack of cooking skill.” (Participant 4800, 31 years) “Lack of meal planning.” (Participant 9915, 35 years)
	Prepare	“Often food preparation is too time-consuming.” (Participant 6493, 18 years)			Poor	“Time poor with 3 young children.” (Participant 3926, 31 years) “Cost is extremely expensive in Wedderburn and quality is poor.” (Participant 9043, 34 years)
	Cost	“Cost from local supermarket is excessive & quality is bad.” (Participant 7361, 42 years)			Busy	“Sometimes skip meals as work so busy.” (Participant 5118, 45 years)
	Work	“Getting home late from work, long work < hours sometimes leads to poor dinner choices.” (Participant 4883, 29 years)			Meals	“I only have 2 meals a day due to working shift work hours.” (Participant 6421, 51 years)
					Day	“Varies each day due to time and availability to me.” (Participant 8302, 20 years)
**Preference and appetite**			Meals	“Time-usually eat one main meal per day.” (Participant 22,906, 59 years)		
			Serves	“I eat a balanced diet, five serves seems a lot per day.” (Participant 8482, 41 years)		
			Day	“I only eat one meal a day.” (Participant 3141, 53 years)		
			Food	“Not hungry enough to eat more food.” (Participant 4013, 24 years) “Have other varieties of food.” (Participant 16,904, 74 years)		
**Meal choices and unachievable guidelines**			Like	“Don’t like them much. They go off too quickly.” (Participant 9935, 45 years) “I eat a balanced diet, five serves seems a lot per day.” (Participant 8481, 41 years)		
			Diet	“I don’t eat 5 serves but I eat a very healthy diet.” (Participant 3164, 67 years)		
**Variety of food, appetite and satisfied with current diet**					Diet	“I like more variety than just veges.” (Participant 22,085, 50 years) “At 69 years my appetite has decreased.” (Participant 12,467, 69 years)
					Serves	“2 serves is ok for me.” (Participant 228,030, 61 years)

Consistent and divergent barriers and the corresponding concepts across genders are summarised in Table 4. Within the theme “cost, quality, and time constraints” in the overall sample, participants reported that cooking was too time consuming which is further exasperated by work commitments and vegetables being too expensive. There was some indication that barriers in females were at the social-environment level, where females stated they did not have enough time to eat vegetables as childcare commitments took priority, and they had a lack of motivation to eat healthy. Whereas in males, individual level barriers of “preference and appetite” and “meal choices and unachievable guidelines” were often noted, with reports that they only ate vegetables once per day, their appetite was too small to consume five serves, their diet was already healthy, and vegetables went off too quickly. Finally, females experienced the barrier “satisfied with current diet” and “variety of food and appetite”. Females expressed that they preferred more variety in their diet than just vegetables and their appetite was too small.

## Discussion

This mixed methods research identified barriers and facilitators to fruit and vegetable consumption across all levels of the socio-ecological framework, which differed between fruit and vegetables as well as by gender. Specifically, lower alcohol intake (an individual level determinant) was a facilitator for meeting fruit recommendations, not living alone (a social-environmental level determinant) was a facilitator for vegetables and community garden use (physical-environmental level determinant) was a facilitator for both fruit and vegetables. For fruit, barriers in males were a preference for other processed snacks or no time to eat fruit at work, alternatively females were concerned with the expense of fruit and stated they inconsistently met the guidelines. Males often described barriers for vegetables as a dislike of the taste and only consuming vegetables at dinner, whilst females believed they could not eat the volume of five serves and preferred a variety of other foods. These results provide insights for potential food group specific and gender specific policies and interventions to improve consumption of both fruit and vegetables in rural Australian communities.

Determinants of fruit and vegetable intake identified in this research were predominantly at the individual level and showed strong socio-economic patterning. This is of particular importance for rural populations, which often include higher proportions of socio-economic disadvantage [2], and are burdened by higher cost of goods and services, such as fresh produce [45]. The lack of association with area level disadvantage in this study aligns with some previous research [12, 15], but contrasts with others [16, 19]. This may have been due to previous research including relatively small sample sizes [19], but is also likely to be due to variation in area-level disadvantage across different rural and regional communities [39]. Nonetheless, research suggests that individuals experiencing socio-economic disadvantage have less healthy diets on average [46], and may not perceive the need to change their behaviours [45]. Furthermore, in line with the present findings, proxies for socio-economic position, such as alcohol and smoking, have been consistently and negatively associated with fruit and vegetable intake [47, 48]. This is likely to be due to clustering of unhealthy behaviours [47, 48], and the high calorie content of alcohol means that it may displace other foods, such as vegetables, that are often consumed at meals [47].

Individual level barriers to fruit and vegetable intake emerging from this research aligned with literature on meal practices [12, 26]. Barriers of a small appetite, the perception that the guidelines were unachievable and time to prepare and cook were comparable with previous research [12, 26]. In two qualitative studies among Australian adults, participants indicated that they only ate vegetables with dinner and were not able to consume the volume of vegetables recommended [12, 26]. Research suggests that grazing or snacking throughout the day is associated with a lower quality diet, where snacks are often high energy discretionary foods rather than fruit [12, 26]. Snacking may thus also impact on the size of subsequent main meals consumed, therefore affecting vegetable consumption [49]. However, there is limited research on this in rural populations. Lastly, the lack of time to prepare and cook vegetables was often described by participants in the present study and other research [12], therefore initiatives to improve knowledge of how to prepare and cook vegetables in a time efficient manner may help facilitate consumption in these settings.

At the social-environmental level, poor financial stability has been linked to lower consumption of both fruit and vegetables [12, 15]. In this study, greater food stability was a facilitator of meeting fruit and vegetable recommendations overall, as well as meeting vegetable recommendations in males and fruit recommendations in females. Previous literature has shown that greater food security positively impacts on healthy food consumption [17, 18, 50, 51], though the survey items used within this study captured only one pillar of food security, food stability, and thus were not sufficient to be comparable to the broader concept of food security [50, 51]. Findings on the role of household size were hard to compare to previous literature. Most literature on household size focusses on overall diet quality scores, which includes scores for fruit and vegetables, rather than intake per se [20, 22, 52]. To the authors knowledge, this study is the first to specifically investigate the association between household size and fruit or vegetable consumption in rural Australia. Therefore, using diet quality scores as a proxy for fruit and vegetable consumption, previous literature has confirmed the present findings by showing that living alone is associated with lower diet quality [22, 52]. Research suggests that not living alone promotes regular and healthier shopping and eating habits [20, 53], which would facilitate a higher consumption of fruit and vegetables.

The present study identified that the theme “meal choices and unachievable guidelines” was only relevant as a barrier to vegetables consumption, but not fruit, and only among men. This is consistent with a qualitative study of 12 adult men, which showed that low cooking motivation and enjoyment led to less sharing of meals [53]. As vegetables are mostly consumed at dinner, an eating occasion often shared with others [54, 55], gender may have a greater effect on meeting vegetable recommendations compared with fruit. Our finding that men (but not women) were more likely to meet fruit recommendations when living in a two-person household supports previous literature on men’s diet quality [56], where men are likely to have a healthier diet when living with a female partner [52, 57]. Future strategies to increase fruit and vegetable intake in males should therefore prioritise social-environmental strategies to increase vegetable intake at meals.

Consistent with this research, previous literature has discussed the challenges of obtaining high quality and healthy food options at a reasonable cost within rural Australia, i.e. barriers at the physical environmental level [14]. While a previous study identified cost and availability as barriers to fruit and vegetable intake in regional Victoria [14], the present study extends this by providing a greater understanding of the barriers specific to each food group, i.e. that cost was a more prominent barrier for vegetables and quality was an important barrier for fruit consumption. Furthermore, we identified that use of community gardens may offer a pathway to improve the cost and availability of fruit and vegetables, which aligns with the larger body of research on the benefits of community gardens [23–25]. Similar to previous research [23–25], community garden use was associated with greater fruit and vegetable consumption. However, of the previous literature, few studies have investigated fruits and vegetables as separate food groups [58, 59] and Litt et al., only investigated vegetable consumption; furthermore, none were stratified by gender [23–25]. Within this study, fruit and vegetable intake in males appeared to be less impacted by the use of community gardens than females, which may be explained by females being more health conscious and willing to access these facilities [23–25]. Furthermore, Barnidge et al., suggests that simply working in a community garden was not enough to increase consumption, and that participants also had to acquire the produce and have the food literacy skills to prepare them [25]. It was also suggested that whilst working within the community created a better support network for eating healthier, [23] the idea of growing and nurturing nature also increased the motivation to prioritise health [24].

This study has many strengths. The separation of fruit and vegetables allowed examination of differences in barriers and facilitators. As most interventions that target fruit and vegetables fail to address low vegetable intake, this research informs the optimal barriers and facilitators to target to achieve improvements in vegetable intake. The use of a mixed methods study enabled a more in-depth understanding of context. Finally, the large sample size and weighted analysis allowed for generalisation to the wider Loddon Campaspe region. Limitations of this study should also be acknowledged. Data on physical activity and food security were either not extensive or not collected using validated tools, limiting their interpretation and comparison to other research. In addition, a small number of barriers were available for investigation at the social and physical environmental level, limiting understanding of the full range of potential barriers. Finally, data were self-reported and cross-sectional, therefore there is the potential for misreporting and social desirability biases. Finally, Leximancer software provides both strengths and limitations that should be acknowledged. As Leximancer removes the need for manual coding of concepts into themes, it enables a quicker and more objective analysis than other thematic analysis approaches that rely on subjective handling of quotes. However, this can also result in themes and concepts that appear to be unexplained as Leximancer does not interpret the meaning of each theme. [42] To overcome this within the present study, quotes were examined to determine the meaning and interpretation of themes and concepts.

Findings from this research have implications for future research. Collection of post-pandemic data could be used for monitoring and surveillance of the population to ensure results remain relevant, which is particularly important in light of the rising cost of living and changes to food systems and communities. [60] Future research should also examine additional social and physical environmental barriers, such as cultural influences and proximity to food outlets, and determine whether findings are comparable across other rural areas of Australia. This would allow for targeted and settings-based strategies to increase consumption.

## Conclusion

This mixed methods research identified that barriers and facilitators to meeting fruit and vegetable recommendations exist across all levels of the socio-ecological framework and differed by food group and by gender. Specifically, at the individual level, lower alcohol consumption was associated with increased fruit consumption. Furthermore, at the social-environmental level, not living alone facilitated vegetable consumption and the use of community gardens was associated with both fruit and vegetable consumption at the physical-environmental level. Contextual findings shows that barriers to fruit consumption were described by males as work commitments, limiting snack time and preferences for processed snacks, while females perceived they inconsistently met the guidelines and fruit was too expensive. In contrast, barriers to vegetable consumption in males revealed a dislike of the taste and only consuming vegetables with dinner, while females reported five serves as too much. As a result, findings from this research show that strategies are required to address individual, social, and physical-level barriers to fruit and vegetable intake in both males and females. Future research should determine if these findings are consistent across other rural Australian communities and whether settings-based interventions are needed.

### Electronic supplementary material

Below is the link to the electronic supplementary material.

Supplementary Material 1

Supplementary Material 2

Supplementary Material 3

Supplementary Material 4

Supplementary Material 5

## Data Availability

The data that support the findings of this study are available from The City of Greater Bendigo but restrictions apply to the availability of these data, which were used under license for the current study, and so are not publicly available. Data are however available from the authors upon reasonable request and with permission of The City of Greater Bendigo.

## Abbreviations

BMI: Body mass index
CI: Confidence intervals
COREQ: Consolidated criteria for reporting qualitative research
IPAQ: International physical activity questionnaire
NCD: Non-communicable disease
OR: Odds ratio
SE: Standard error
SEIFA: Socio-Economic Indexes for Areas
STROBE-nut: Strengthening the Reporting of Observational studies in Epidemiology ? Nutritional Epidemiology
