# Supplementary material for: Identifying the barriers and facilitators to fruit and vegetable consumption in rural Australian adults: a mixed methods analysis
Source: Nutr J. 2024 Jun 28;23:69. doi: 10.1186/s12937-024-00972-y (PMC11214237; doi:10.1186/s12937-024-00972-y)
Supplement: Supplementary file 2 — Supplementary Material 2 [file 12937_2024_972_MOESM2_ESM.docx]

**Additional File 2**. Consolidated criteria for reporting qualitative research (COREQ)

| **No** | **Item** | **Guide questions/description** | **Page** |
| --- | --- | --- | --- |
| **Domain 1: Research team and reflexivity** |  |  |  |
| Personal Characteristics |  |  |  |
| 1. | Interviewer/facilitator | Which author/s conducted the interview or focus group? | N/A |
| 2. | Credentials | What were the researcher's credentials? *E.g., PhD, MD* | N/A |
| 3. | Occupation | What was their occupation at the time of the study? | N/A |
| 4. | Gender | Was the researcher male or female? | N/A |
| 5. | Experience and training | What experience or training did the researcher have? | N/A |
| Relationship with participants |  |  |  |
| 6. | Relationship established | Was a relationship established prior to study commencement? | N/A |
| 7. | Participant knowledge of the interviewer | What did the participants know about the researcher? e*.g., personal goals, reasons for doing the research* | N/A |
| 8. | Interviewer characteristics | What characteristics were reported about the interviewer/facilitator? e.g., *Bias, assumptions, reasons and interests in the research topic* | N/A |
| **Domain 2: study design** |  |  |  |
| Theoretical framework |  |  |  |
| 9. | Methodological orientation and Theory | What methodological orientation was stated to underpin the study? *e.g., grounded theory, discourse analysis, ethnography, phenomenology, content analysis* | 8 |
| Participant selection |  |  |  |
| 10. | Sampling | How were participants selected? *e.g., purposive, convenience, consecutive, snowball* | 6-8 |
| 11. | Method of approach | How were participants approached? e*.g., face-to-face, telephone, mail, email* | 5-6 |
| 12. | Sample size | How many participants were in the study? | 5-6 |
| 13. | Non-participation | How many people refused to participate or dropped out? Reasons? | 5-6 |
| Setting |  |  |  |
| 14. | Setting of data collection | Where was the data collected? e*.g., home, clinic, workplace* | 5-6 |
| 15. | Presence of non-participants | Was anyone else present besides the participants and researchers? | N/A |
| 16. | Description of sample | What are the important characteristics of the sample? *e.g., demographic data, date* |  |
| Data collection |  |  |  |
| 17. | Interview guide | Were questions, prompts, guides provided by the authors? Was it pilot tested? | N/A |
| 18. | Repeat interviews | Were repeat interviews carried out? If yes, how many? | N/A |
| 19. | Audio/visual recording | Did the research use audio or visual recording to collect the data? | N/A |
| 20. | Field notes | Were field notes made during and/or after the interview or focus group? | N/A |
| 21. | Duration | What was the duration of the interviews or focus group? | N/A |
| 22. | Data saturation | Was data saturation discussed? | N/A |
| 23. | Transcripts returned | Were transcripts returned to participants for comment and/or correction? | N/A |
| **Domain 3: analysis and findings** |  |  |  |
| Data analysis |  |  |  |
| 24. | Number of data coders | How many data coders coded the data? | 9 |
| 25. | Description of the coding tree | Did authors provide a description of the coding tree? | 8-9 |
| 26. | Derivation of themes | Were themes identified in advance or derived from the data? | 8-9 |
| 27. | Software | What software, if applicable, was used to manage the data? | 8 |
| 28. | Participant checking | Did participants provide feedback on the findings? | N/A |
| Reporting |  |  |  |
| 29. | Quotations presented | Were participant quotations presented to illustrate the themes / findings? Was each quotation identified? e*.g., participant number* | Table 3 & Table 4 |
| 30. | Data and findings consistent | Was there consistency between the data presented and the findings? | 13-14 |
| 31. | Clarity of major themes | Were major themes clearly presented in the findings? | Additional File 5 |
| 32. | Clarity of minor themes | Is there a description of diverse cases or discussion of minor themes? | 13-14 |
