# Supplementary material for: Identifying the barriers and facilitators to fruit and vegetable consumption in rural Australian adults: a mixed methods analysis
Source: Nutr J. 2024 Jun 28;23:69. doi: 10.1186/s12937-024-00972-y (PMC11214237; doi:10.1186/s12937-024-00972-y)
Supplement: Supplementary file 3 — Supplementary Material 3 [file 12937_2024_972_MOESM3_ESM.docx]

**Additional File 3.** Original questions and response options and groupings used for quantitative analysis.

| **Question** | **Original response options** | **Groupings for logistic regression** |
| --- | --- | --- |
| A3. What is the postcode of your residence? | Open ended response of 4 digits | 1 – most disadvantaged (area level disadvantage) |
|  |  | 2 |
|  |  | 3 |
|  |  | 4 |
|  |  | 5 – least disadvantaged (area level disadvantage) |
| A4. Including yourself, how many people aged 3 years and over currently live in your household? | Open ended response of 2 digits | 1 person |
|  |  | 2 people |
|  |  | 3+ people |
| A5. Given your current needs and financial responsibilities, would you say that you and your household are…? | Prosperous | Prosperous/Very comfortable |
|  | Very comfortable |  |
|  | Reasonable comfortable | Reasonably comfortable |
|  | Just getting along |  |
|  | Poor | Just getting along/Poor/Very poor |
|  | Very poor |  |
| A6. In the past 12 months, was there any time when your household ran out of food and could not afford to buy more? | Yes | Yes |
|  | No | No |
| B1. How old are you? | Open ended response of 3 digits | Continuous variable of up to 3 digits |
| B2. Which of the following best describes your current gender identity? | Male | Male |
|  | Female | Female |
|  | Gender diverse / Non-binary / Self-described / Other | Gender diverse *(removed for stratification)* |
| B7. What is the highest level of education you have completed? | Tertiary degree or higher | Tertiary degree or higher |
|  | Completed year 12 | Completed year 12 |
|  | Have not finished year 12 / still in school | Have not completed year 12 |
| C3. Which of the following types of public facilities or open spaces have you used in your area in the last 12 months? | Community gardens | Community gardens |
|  | Yes | Yes |
|  | No | No |
| E4. How many serves of vegetables, legumes or beans do you usually eat each day? | Open ended question to one decimal point | Meeting the daily recommended intake^1^ |
|  |  | Yes |
|  |  | No |
| E5. How many serves of fruit do you usually eat each day? | Open ended question to one decimal point | Meeting the daily recommended intake^2^ |
|  |  | Yes |
|  |  | No |
| E8. How many cups of water do you usually drink in a day? | Open ended question in cups per day to one decimal point | Continuous variable in cups per day |
| E9. How often do you consume cordial, soft drinks, flavoured mineral water, energy or sports drinks…? | Every day | More than once per week |
|  | Several times per week |  |
|  | About once a week | Once per week/fortnight |
|  | About once a fortnight |  |
|  | About once a month | Once or less per month |
|  | Less often than once pre month |  |
|  | Never | Never |
| E10. How tall are you without shoes? | Open ended response in centimetres (cm) or feet (ft)/inches (inch) | BMI^3^ |
|  |  | Underweight/Normal weight (<25 kg/m^2^) |
| E11. What is your weight without clothes or shoes? | Open ended response in kilograms (kg) or stones (st)/pounds (lb) | Pre-obesity (25-30 kg/m^2^) |
|  |  | Obesity (>30 kg/m^2^) |
| E13. Excluding household chores and gardening, in the last week, what do you estimate was the total time that you spent doing vigorous physical activity (e.g. tennis, jogging, cycling or keep fit exercises) that made you breathe harder or puff and pant? | Open ended response in minutes per day | Continuous variable in hours per day |
| E15. Which of the following best describes your smoking status? | Smoke daily | Current smoker |
|  | Smoke occasionally |  |
|  | Don’t smoke now, but used to | Used to smoke |
|  | Tried a few times but never smoked regularly |  |
|  | Never smoked | Never smoked |
| E16. In the last 12 months, how often did you have an alcoholic drink of any kind? | Every day | More than 3 days per week  1-2 days per week |
|  | 3 to 6 days a week |  |
|  | 1 to 2 days a week | 1-2 days per week |
|  | 1 to 3 days a month |  |
|  | Less than once a month | 3 or less days per month |
|  | No longer drink |  |
|  | Do not drink | No longer drink/do not drink |

1, The recommended vegetable intake was considered more than five serves per day. (5) 2, The recommended fruit intake was considered more than two serves per day. (5) 3, Weight status categories were determined based on BMI cut offs from the World Health Organisation. (43)
