# Supplementary material for: Identifying the barriers and facilitators to fruit and vegetable consumption in rural Australian adults: a mixed methods analysis
Source: Nutr J. 2024 Jun 28;23:69. doi: 10.1186/s12937-024-00972-y (PMC11214237; doi:10.1186/s12937-024-00972-y)
Supplement: Supplementary file 4 — Supplementary Material 4 [file 12937_2024_972_MOESM4_ESM.docx]

**Additional File 4**. Socio-demographic and anthropometric characteristics overall and by gender from the Active Living Census 2019 included in the analysis (complete case analysis) and those excluded from the anlaysis.^1^

| **Characteristics** | **Analytical sample (n=13464)** | **Excluded sample (n=7430)^2^** |
| --- | --- | --- |
| Age (years), n, mean (SE) | 48.0 (0.17) | 50.30 (0.31) |
| Age groups, n (%) |  |  |
| 18-30 years | 2599 (19.3) | 1070 (15.4) |
| 31-50 years | 4739 (35.2) | 1827 (26.3) |
| 51-70 years | 4685 (34.8) | 2556 (36.8) |
| >70 years | 1441 (10.7) | 1494 (21.5) |
| Gender, n (%) |  |  |
| Male | 6557 (48.7) | 3222 (47.4) |
| Female | 6867 (51.0) | 3562 (52.4) |
| Other | 31 (0.23) | 7 (0.001) |
| BMI (kg/m^2^), mean (SE) | 27.4 (0.06) | 27.5 (0.10) |
| Weight status, n (%)^3^ |  |  |
| Underweight/Normal weight (<25 kg/m^2^) | 5157 (38.3) | 1602 (21.9) |
| Pre-obesity (25-30 kg/m^2^) | 4780 (35.5) | 1631 (22.3) |
| Obesity (>30 kg/m^2^) | 3528 (26.3) | 4081 (55.8) |
| Area level disadvantage, n (%) |  |  |
| 1 – most disadvantaged | 1912 (14.2) | 1198 (16.5) |
| 2 | 5709 (42.4) | 3209 (44.2) |
| 3 | 808 (6.60) | 503 (6.93) |
| 4 | 3191 (23.7) | 1474 (20.3) |
| 5 – least disadvantaged | 1764 (13.1) | 871 (12.0) |

1, Values represented weight mean and standard errors (SE) for continuous variables and weighted frequencies for categorical variables. 2, The total excluded sample was 7,430, however this sample was not the same for each variable as the data missing differed between variables. 3, Weight status categories were determined based on BMI cut offs from the World Health Organisation. (43)
