# Supplementary material for: Identifying the barriers and facilitators to fruit and vegetable consumption in rural Australian adults: a mixed methods analysis
Source: Nutr J. 2024 Jun 28;23:69. doi: 10.1186/s12937-024-00972-y (PMC11214237; doi:10.1186/s12937-024-00972-y)
Supplement: Supplementary file 5 — Supplementary Material 5 [file 12937_2024_972_MOESM5_ESM.docx]

**Additional File 5**. Leximancer ranked theme and concepts list of the barriers to fruit and vegetable consumption for the overall sample.

| **Fruit** | | **Vegetable** | |
| --- | --- | --- | --- |
| **Theme** | **Concept** | **Theme** | **Concept** |
| EAT | eat | TIME | time |
|  | fan |  | meals |
|  | like |  | cook |
|  | food |  | food |
|  | day |  | prepare |
|  | serves |  | cost |
|  | forget |  | work |
|  | feel |  | lack |
|  | prefer |  | busy |
|  | buy |  | poor |
|  | busy |  | lazy |
|  | vegetables |  | home |
|  | need |  | taste |
|  | hungry |  | buy |
|  | meals |  | convenience |
|  | snack |  | fresh |
|  | season |  | difficult |
|  | depends |  | quality |
|  | usually |  | working |
|  | summer |  | due |
|  | piece |  | available |
|  | enjoy | EAT | eat |
|  | daily |  | day |
|  | winter |  | like |
| TIME | time |  | serves |
|  | cost |  | eating |
|  | work |  | feel |
|  | poor |  | need |
|  | lack |  | fruit |
|  | expensive |  | lunch |
|  | health |  | healthy |
|  | quality |  | depends |
|  | afford |  | dinner |
|  | money |  | fit |
|  | taste |  | usually |
|  | availability |  | try |
|  | lazy |  | main |
|  | fresh |  | breakfast |
|  | available |  | large |
|  | bad | DIET | diet |
|  | variety |  | expensive |
| SUGAR | sugar |  | variety |
|  | diet |  | health |
|  | fructose |  | afford |
|  | try |  | eater |
|  | intake |  | experts |
|  | low | MEAT | meat |
|  | sweet |  | hungry |
|  | due |  | salad |
| SEASONAL | seasonal | MONEY | money |
|  | eater | APPETITE | appetite |

1. **males**
2. **males**

**(b) females**
